# *AP3S1* is a Novel Prognostic Biomarker and Correlated With an Immunosuppressive Tumor Microenvironment in Pan-Cancer

**DOI:** 10.3389/fcell.2022.930933

**Published:** 2022-07-08

**Authors:** Gujie Wu, Mianxiong Chen, Hefei Ren, Xinyu Sha, Min He, Kuan Ren, Juntao Qi, Feng Lin

**Affiliations:** ^1^ Department of Urology, Shenzhen Traditional Chinese Medicine Hospital, Guangzhou University of Chinese Medicine, Shenzhen, China; ^2^ Research Center of Clinical Medicine, Affiliated Hospital of Nantong University, Nantong, China; ^3^ Department of Laboratory Medicine, Changzheng Hospital, Naval Medical University, Shanghai, China

**Keywords:** AP3S1, pan-cancer, prognostic biomarker, immunosuppressive microenvironment, immunotherapy

## Abstract

**Background:** Adaptor-related protein complex 3, sigma one subunit (AP3S1) is one of the encoding subunits of the adaptor complex AP-3. However, its role in various tumor types and relationship with the tumor immune microenvironment (TIME) remains unclear.

**Methods:** AP3S1 expression was analyzed using datasets from The Cancer Genome Atlas, Genotype-Tissue Expression, UALCAN, and HPA databases. Then, we performed a systematic analysis of the genetic alterations, clinical features, and prognostic value of AP3S1 in pan-cancer. Gene set enrichment analysis (GSEA) and gene set variation analysis (GSVA) were used to identify the signaling pathways associated with AP3S1. The correlation between immune cell infiltration and AP3S1 expression was analyzed using immune cell infiltration data from the ImmuCellAI, TIMER2, and a previous study. Finally, we analyzed the association of AP3S1 with tumor mutational burden (TMB), microsatellite instability (MSI), and immune-related genes.

**Results:** We found AP3S1 overexpression in most tumors and a significant association with low survival rates. GSEA and GSVA results show that AP3S1 is involved in tumor progression and associated with immune pathways in different tumor types. We also found that AP3S1 expression was positively correlated with the level of infiltration of immunosuppressive cells (tumor-associated macrophages, cancer-associated fibroblasts, Tregs) and negatively correlated with immune killer cells, including NK cells and CD8^+^ T cells, in pan-cancer. The expression of AP3S1 could affect TMB and MSI in various cancers. In addition, AP3S1 was positively correlated with most immunosuppressive genes, including PD-1, PD-L1, CTLA4, LAG3 and TIGIT in most cancer types.

**Conclusion:** Our study reveals that AP3S1 is a potential pan-cancer oncogene and plays an essential role in tumorigenesis and cancer immunity. Elevated expression of AP3S1 indicates an immunosuppressive microenvironment and can be used as a potential prognostic biomarker and a target for immunotherapy.

## Introduction

Cancer is the leading cause of death globally and seriously impacts people’s survival (Siegel et al., 2020). Although tremendous progress has been made in various treatments for tumours in recent years, the prognosis and survival rates for many types of cancers remain unsatisfactory (Kandoth et al., 2013; Zhang et al., 2020; He et al., 2021). Growing evidence suggests that the immunomodulatory mechanisms of tumors have a notable impact on tumor growth, progression, tumor microenvironment and patient prognostic outcome (Lei et al., 2020; Li et al., 2016; Mittal et al., 2018). Therefore, given the promising potential of the tumor immune microenvironment (TIME) in cancer therapy, there is an urgent need to explore the underlying mechanisms of tumors and to identify novel critical biomarkers for tumor patients.

AP3S1 encodes a subunit of the AP-3 complex, an adapter-related complex that is associated with the Golgi apparatus and more peripheral structures (Simpson et al., 1997). To date, only a few studies have reported a role for AP3S1 in the development of tumors. A recent study identified AP3S1 as a tumor driver gene after analyzing 1,145 samples from patients with esophageal squamous cell carcinoma (Zou et al., 2021). Additionally, AP3S1 expression is associated with the progression of rectal and cervical tumors (Petrenko et al., 2006; Nome et al., 2013). Despite these findings, additional studies are needed to reveal more about the role of AP3S1 in tumor development and progression. Our study explores the potential pan-cancer role of AP3S1 through an extensive analysis and its function in the tumour microenvironment (TME).

In this study, we used multi-omics data from 33 cancers to comprehensively analyse the role of AP3S1, including AP3S1 expression, clinical characteristics, prognostic role, DNA methylation, copy number alteration (CNA), and mutational status. GSEA and GSVA results show that AP3S1 is involved in tumor progression and associated with immune pathways in various tumors. Finally, we assessed the correlation between AP3S1 expression, immune cell infiltration levels and immune-related genes. Elevated AP3S1 expression predicts a tumor immunosuppressive microenvironment. Our findings reveal an essential role for AP3S1 in the tumour immunosuppressive microenvironment and AP3S1 could act a potential prognostic and immunotherapeutic biomarker.

## Materials and Methods

### Data Source

Our RNAseq data and clinical information data were derived from The Cancer Genome Atlas (TCGA) and Genotype-Tissue Expression (GTEx). These data are available for download from the UCSC Xena (xenabrowser.net/datapages/) database. cBioPortal database (www.cbioportal.org/) provided us with AP3S1 methylation and CNA data. The Human Protein Atlas database (www.proteinatlas.org/) was used to explore the protein level of AP3S1 in human tumors and normal tissues.

### Prognostic Analysis

Kaplan-Meier analysis was performed to evaluate the overall survival (OS) of patients from TCGA cohort. Univariate Cox regression analyses were conducted to assess the significance of AP3S1 in predicting OS, disease-specific survival (DSS), the disease-free interval (DFI), and the progression-free interval (PFI) in pan-cancer.

### GSEA and GSVA

GSEA analysis was used to evaluate AP3S1 and all associated genes in pan-cancer. Pearson correlation coefficients and the R package “clusterProfiler” were used in the process. GSVA was performed by the “ssGSEA” algorithm and the data were obtained from the MSigDB database by downloading the landmark pathways.

### Immune Infiltration Analysis

The R package “ESTIMATE” was used to calculate the stromal score, immune score, and tumor purity score of each patient in TCGA cohort. The association between AP3S1 expression and these scores was analyzed. The TME-related pathways were obtained and pathway scores were calculated according to the published paper (Zeng et al., 2019). The immune cell infiltration data were downloaded from Immune Cell Abundance Identifier (ImmuCellAI) database (http://bioinfo.life.hust.edu.cn/ImmuCellAI#!/) and TIMER2 database (http://timer.cistrome.org/).

### Statistical Analysis

All data used in this article are presented as the mean ± standard deviation (SD). The correlation analysis between the two variables used Spearman's or Pearson's test. Statistical analysis was performed using R v4.1.1. A *p*-value < 0.05 was considered statistically significant.

## Results

### Expression of AP3S1 in Pan-Cancer

**FIGURE 1:**
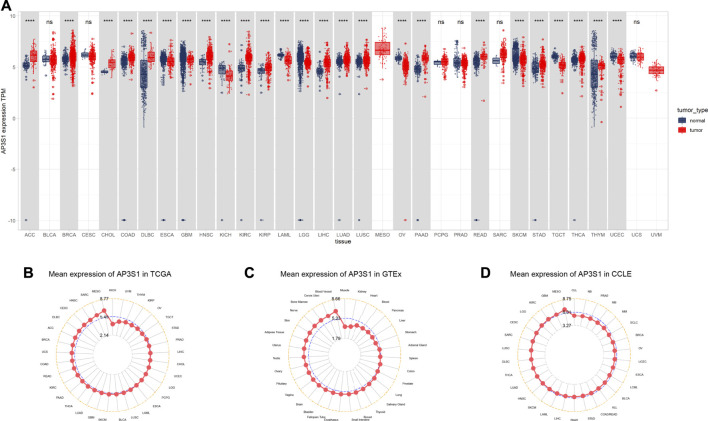
Expression of AP3S1 in pan-cancer. **(A)** pan-cancer expression of AP3S1. **(B)** AP3S1 expression in tumor tissues from TCGA cohort. **(C)** AP3S1 expression in normal tissues from GTEx cohort. **(D)** AP3S1 expression in cancer cell lines from the CCLE cohort.*p < 0.05, **p < 0.01, ***p < 0.001, ****p < 0.0001.

Firstly, we assessed AP3S1 expression using TCGA and GTEx data. We found that AP3S1 was overexpressed in 16 of 33 cancer types, including Adrenocortical carcinoma (ACC), Breast invasive carcinoma (BRCA), Cholangiocarcinoma (CHOL), Colon adenocarcinoma (COAD), Lymphoid Neoplasm Diffuse Large B-cell Lymphoma (DLBC), Head and Neck squamous cell carcinoma (HNSC), Kidney renal clear cell carcinoma (KIRC), Kidney renal papillary cell carcinoma (KIRP), Liver hepatocellular carcinoma (LIHC), Lung adenocarcinoma (LUAD), Lung squamous cell carcinoma (LUSC), Pancreatic adenocarcinoma (PAAD), Rectum adenocarcinoma (READ), Stomach adenocarcinoma (STAD), Thyroid carcinoma (THCA) and Thymoma (THYM). Moreover, the expression of AP3S1 was found to be significantly reduced in Esophageal carcinoma (ESCA), Glioblastoma multiforme (GBM), Kidney Chromophobe (KICH), Acute Myeloid Leukemia (LAML), Lower Grade Glioma (LGG), Ovarian serous cystadenocarcinoma (OV), Skin Cutaneous Melanoma (SKCM), Testicular Germ Cell Tumor (TGCT), Uterine Corpus Endometrial Carcinoma (UCEC) (Figure 1A and Supplementary Table S1). In order to compare AP3S1 expression in tumor tissues, our findings revealed that AP3S1 expression was highest in MESO and lowest in KICH (Figure 1B). In normal tissues from GTEx database, the results revealed that AP3S1 expression was the highest in blood vessel tissues and the lowest in muscles (Figure 1C). As for tumor cell lines, we proved that AP3S1 expression was also the highest in MESO cell lines using data from CCLE database (Figure 1D).

**FIGURE 2:**
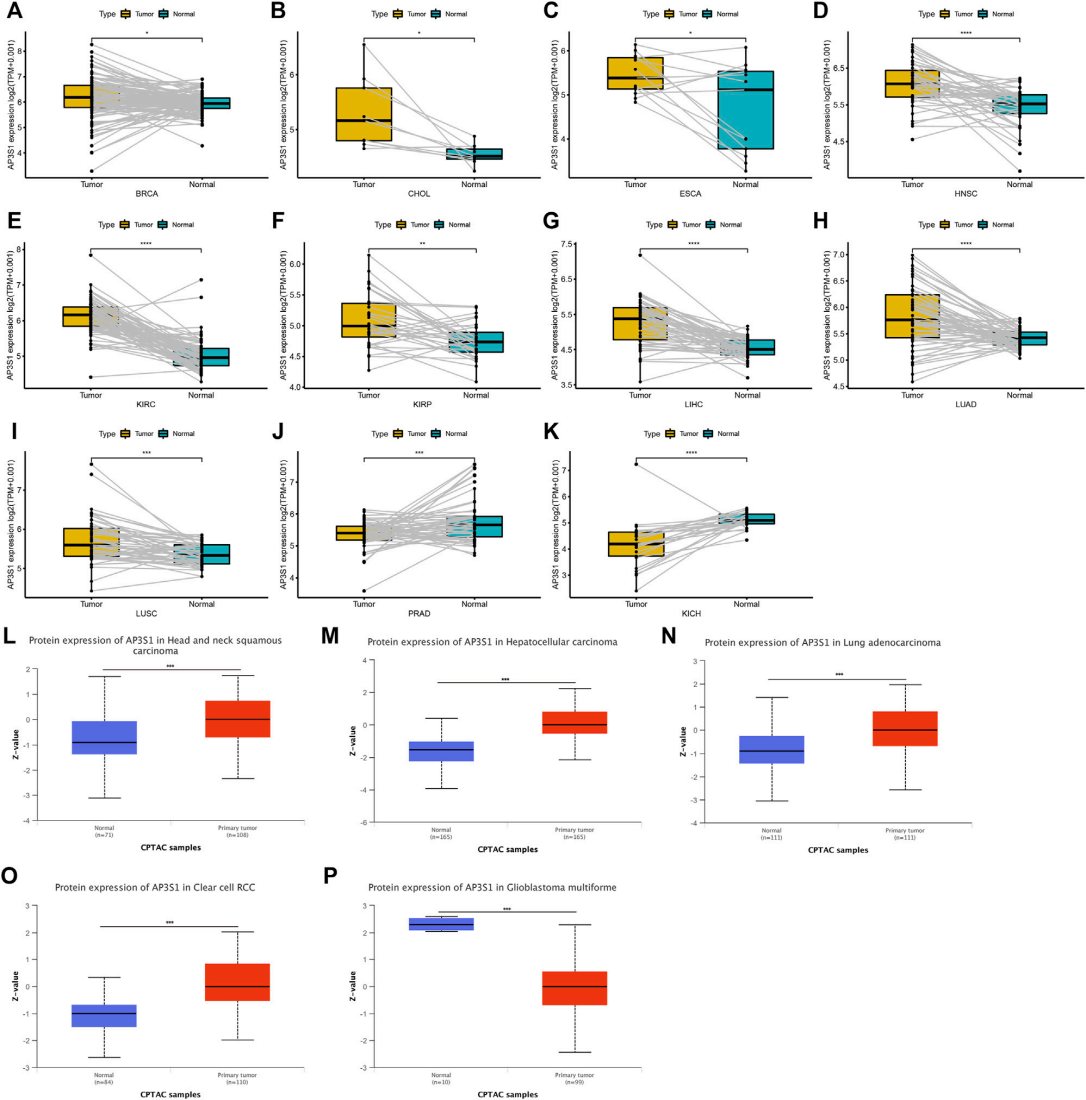
Expression of AP3S1 in paired tumor and adjacent normal tissues. **(A–K)** AP3S1 expression in paired tumor and adjacent normal tissues from TCGA in indicated tumor types. **(L–P)** The protein level of AP3S1 in indicated tumor types using Ualcan database*p < 0.05, **p < 0.01, ***p < 0.001, ****p < 0.0001.

AP3S1 was also observed to be overexpressed in 10 cancers such as BRCA, CHOL, ESCA, HNSC, KIRC, KIRP, LIHC, LUAD, and LUSC by comparing tumors with adjacent normal tissues in the TCGA cohort (Figures 2A–I), with lower expression only in PRAD and KICH (Figures 2J,K). After analysis of the UALCAN database, AP3S1 protein was upregulated in tumor tissues of HNSC, LIHC, LUAD, and KIRC and downregulated in GBM compared to normal tissues (Figures 2L–P). The HPA database of protein levels of AP3S1 expression between normal and tumor tissues also confirms similar results (Supplementary Figure S1). Additionally, we investigated AP3S1 expression at various tumor stages. The results demonstrated that AP3S1 expression was elevated in the relatively worse tumor stages in ACC, CESC, HNSC, KIRP, LUAD, PAAD, and THCA (Supplementary Figure S2).

### Gene Alteration of AP3S1 in Pan-Cancer

**FIGURE 3:**
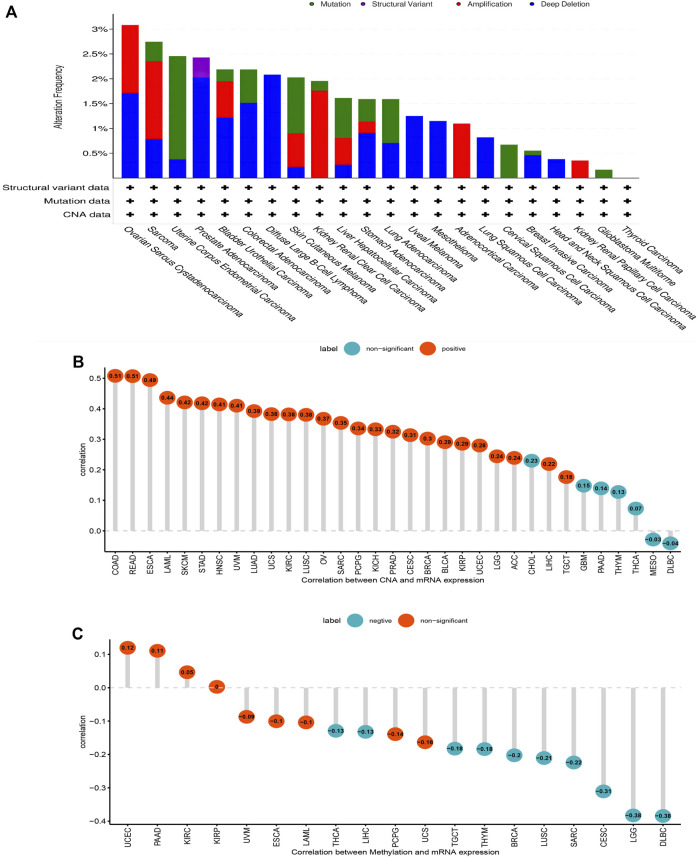
Gene alteration of AP3S1. **(A)** The mutation and CNA status of AP3S1 in TCGA-pan-cancer. **(B)** The correlation between AP3S1 expression and CNA. **(C)** The correlation between AP3S1 expression and DNA methylation.

Gene mutations, DNA methylation, and CNA are closely associated with tumor development and progression. Our analysis of AP3S1 gene alterations using cBioPortal showed that the highest alteration frequency of AP3S1 appears for patients in uterine corpus endometrial carcinoma, with “amplification and deep deletion” as the primary types (Figure 3A). By correlation analysis between AP3S1 and CNA, we found that AP3S1 expression was positively correlated with CNA in most tumor types (Figure 3B). DNA methylation as an epigenetic modification usually leads to the silencing or inactivation of tumor suppressor genes, which results in cancer development and proliferation. Therefore, we further calculated the correlation levels between AP3S1 and promoter methylation. Gene expression significantly correlated with the methylation status in 19 tumors, with the highest negative correlations in LGG and DLBL (Figure 3C).

### Prognostic Role of AP3S1

**FIGURE 4:**
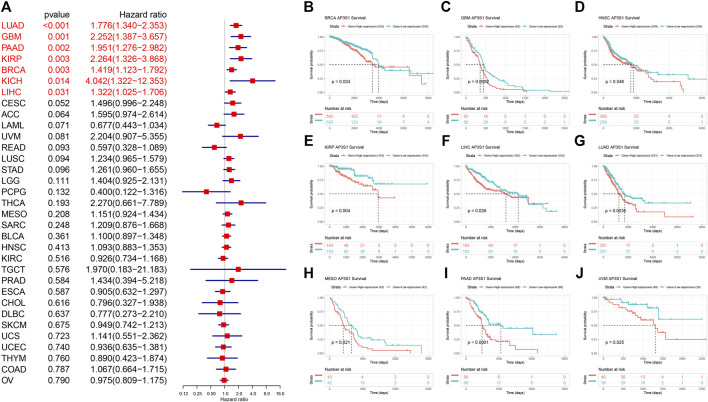
Relationship between AP3S1 level and OS. **(A)** The univariate Cox regression OS analysis of AP3S1 in TCGA pan-cancer. Red color represents significant results (*p* < 0.05). **(B–J)** Kaplan-Meier curves showing OS in pan-cancer.

**FIGURE 5:**
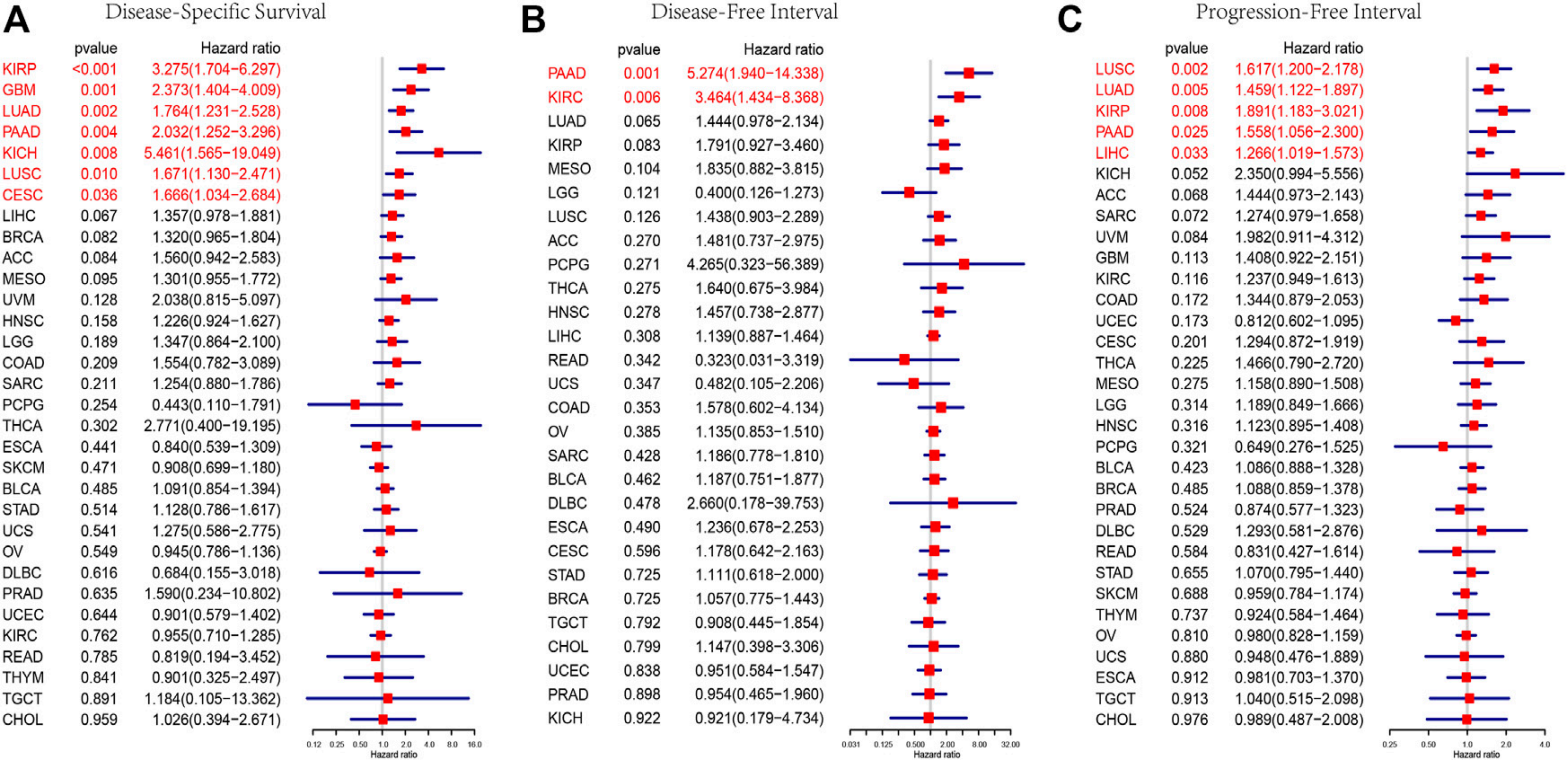
Prognostic value of AP3S1. Forest plots showing results of Univariate Cox Regression analysis for **(A)** DSS, **(B)** DFI, and **(C)** PFI. Red color represents significant results (*p* < 0.05).

To further explore the impact of AP3S1 expression in pan-cancer on patient prognosis, we performed survival analysis of AP3S1 expression and patient survival data using univariate Cox regression analysis (UniCox) and Kaplan-Meier methods. The analysis revealed that AP3S1 was a risk factor for LUAD, GBM, PAAD, KIRP, BRCA, KICH and LIHC (Figure 4A). Kaplan-Meier OS analysis demonstrated that elevated AP3S1 expression predicts BRCA, GBM, HNSC, KIRP, LIHC, LUAD, MESO, PAAD and UVM patients with poor overall survival (OS) (Figures 4B–J). Disease-specific survival (DSS) analysis showed AP3S1 as a risk factor in KIRP, GBM, LUAD, PAAD, KICH, LUSC and CESC (Figure 5A). Moreover, a high AP3S1 expression predicted a shorter disease-free interval (DFI) in patients with PAAD and KIRC (Figure 5B). At the same time, a high AP3S1 expression predicted a worse progression-free interval (PFI) in patients with LUSC, LUAD, KIRP, PAAD and LIHC (Figure 5C).

### GSEA and GSVA of AP3S1

**FIGURE 6:**
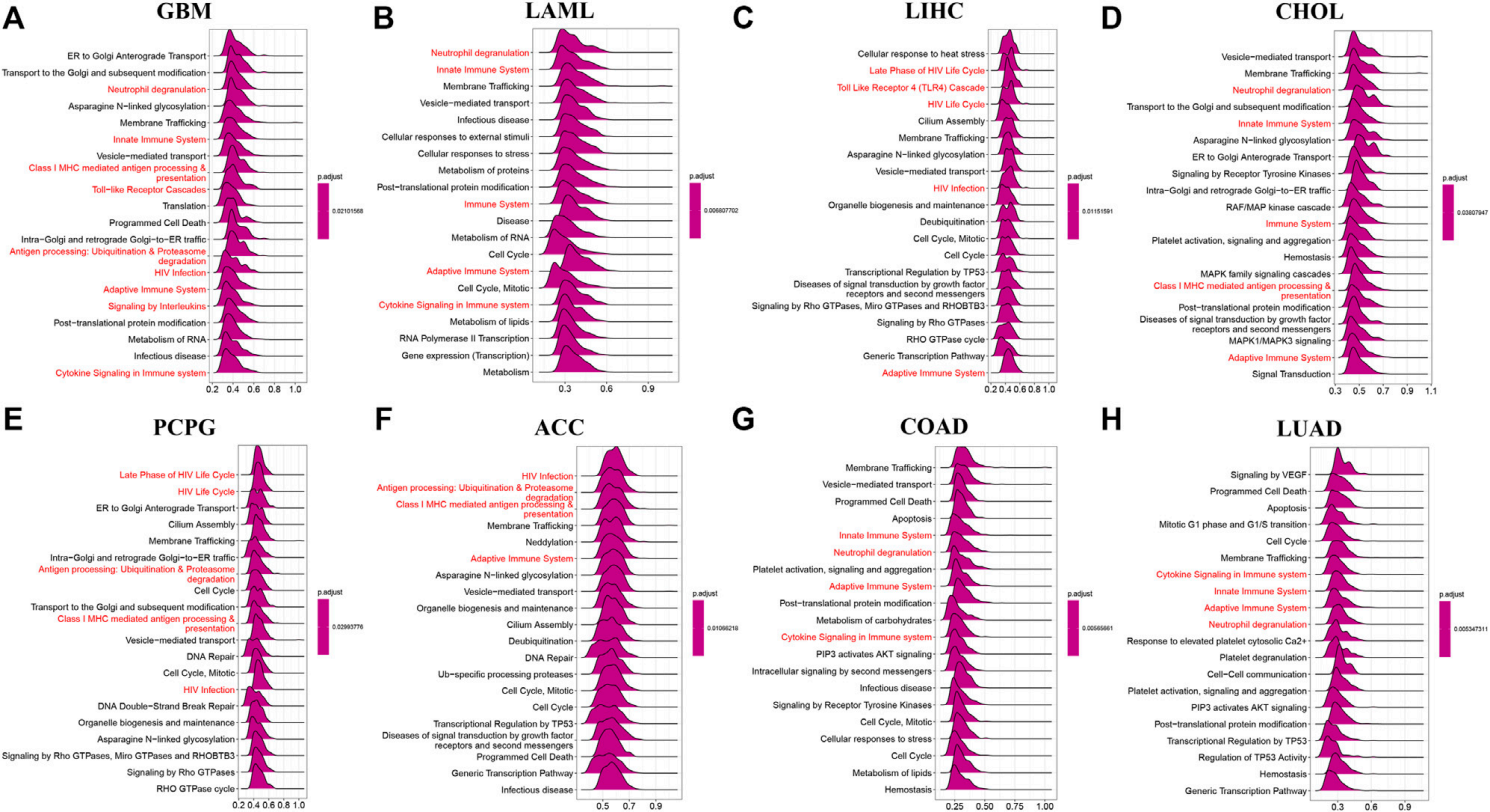
GSEA of AP3S1 in TCGA pan-cancer. **(A–H)** The top 8 significant pathways of AP3S1 GSEA results across the indicated tumor types. Red color represents immune-related pathways.

In order to elucidate the potential biological pathways that AP3S1 might regulate, we performed a GSEA algorithm analysis using the “clusterprofiler” in pan-cancers and then selected 8 tumors with similar results (Figures 6A–H). We found that AP3S1 participates in immune regulation-related pathways in pan-cancer, especially for the adaptive and innate immune systems, neutrophil degranulation, HIV infection, cytokine signalling in the immune system, antigen processing and Toll-like receptor cascades. In addition, cycle-related pathways (e.g. “cell cycle,” “ER to Golgi anterograde transport” and “vesicle-mediated transport”) were closely associated with AP3S1. These results indicated that AP3S1 plays a significant role in tumor development and tumor immunity.

**FIGURE 7:**
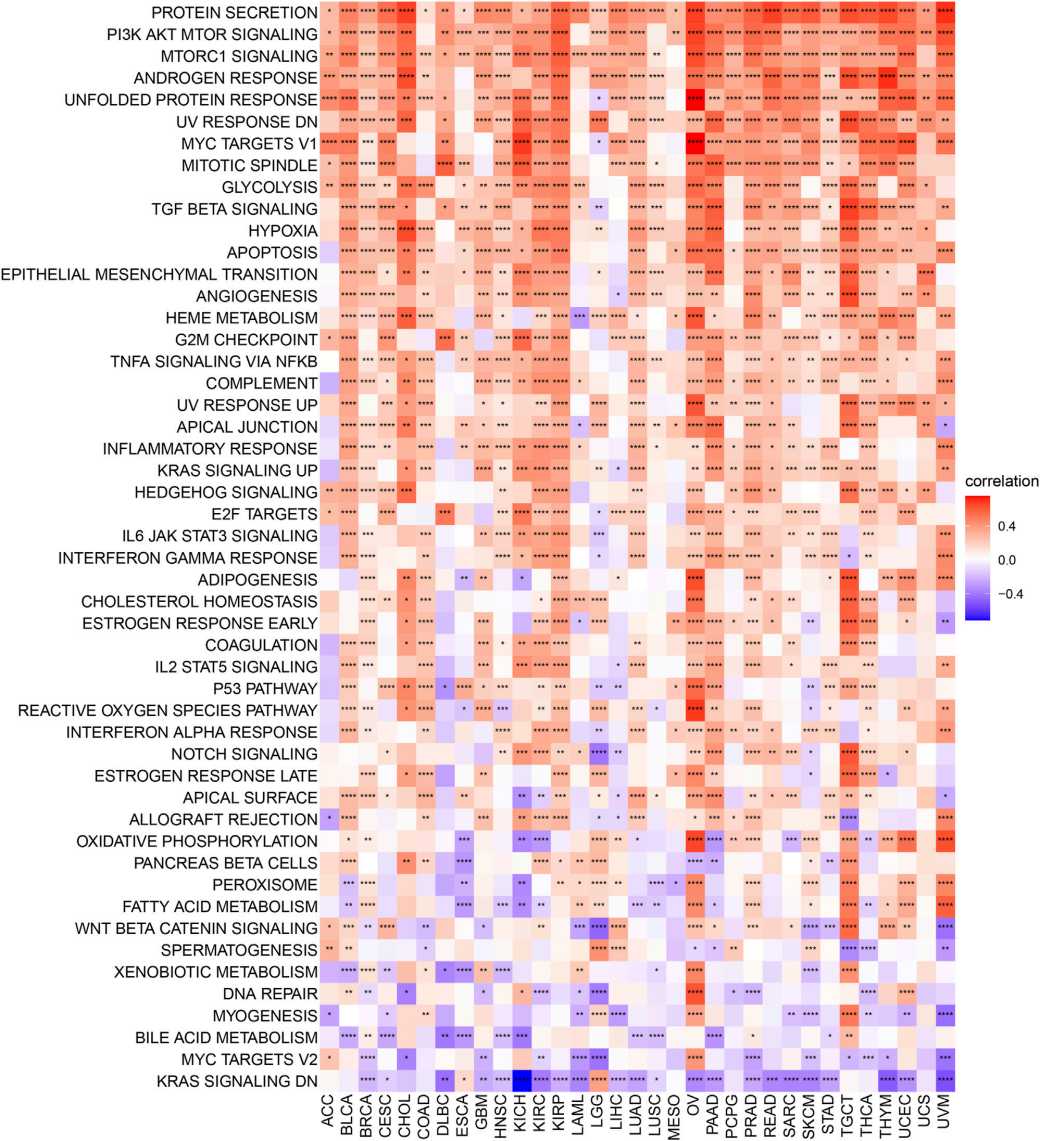
GSVA of AP3S1 in TCGA pan-cancer. GSVA results of 50 hallmark pathways from the MSigDB.

To analyse the potential pathways affected by the AP3S1 expression, we performed a GSVA based on 50 HALLMARK pathways. The association between the AP3S1 expression and GSVA scores in pan-cancer is shown in Figure 7. We observed that the AP3S1 overexpression was positively associated with many malignant pathways in pan-cancer, such as IL6 JAK STAT3 signaling, IL2 STAT5 signaling, TGF BETA signaling, interferon gamma response, and interferon alpha response. All these pathways were also closely associated with TIME.

### Immune Cell Infiltration Analysis

**FIGURE 8:**
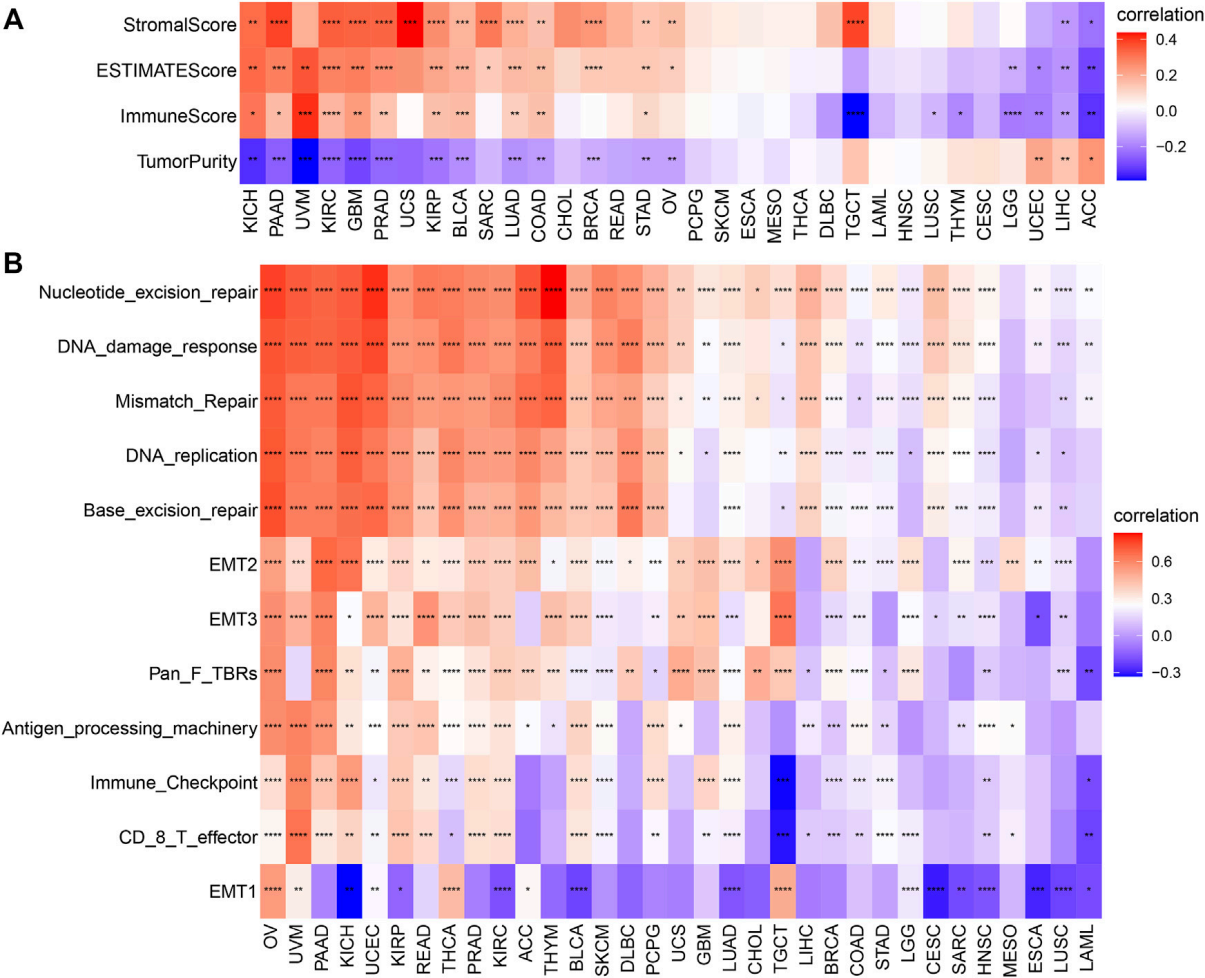
The relationship between AP3S1 and the regulation of the tumour microenvironment. **(A)** Heatmap represents the correlation between AP3S1 expression and TME scores in pan-cancer. **(B)** The relationship between AP3S1 and the tumour microenvironment. Red represents positive correlation, blue represents negative correlation, and the darker the color, the stronger the correlation. *p < 0.05, **p < 0.01, ***p < 0.001, ****p < 0.0001.

Subsequently, we further assessed the association between AP3S1 expression and stromal and immune scores using the “ESTIMATE” algorithm (Figure 8A). The results showed that AP3S1 expression was able to influence immune scores, stromal scores and ESTIMATE scores in most tumors. To validate this finding, we obtained further data and calculated the TME-related pathways involved in AP3S1, including immune-related pathways, stroma-related pathways and DNA repair-related pathways, based on published papers. The results also showed that AP3S1 expression was closely associated with immune-related pathways, including immune checkpoints, antigen processing mechanisms and CD8 T effectors in pan-cancer (Figure 8B).

**FIGURE 9:**
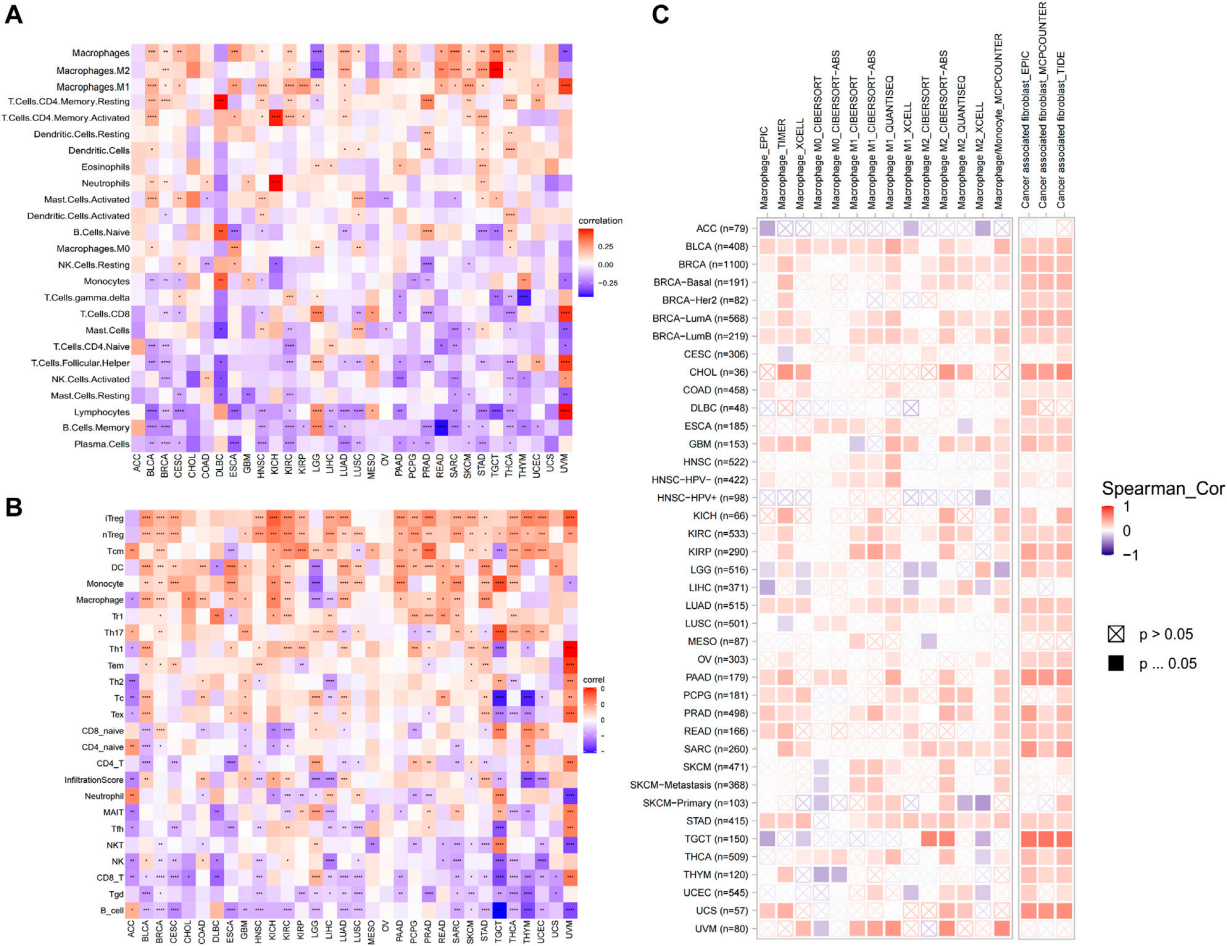
The relationship between AP3S1 and the immune cell infiltration. **(A)** Correlation between AP3S1 and different immune cells from previous study. **(B)** Correlation between AP3S1 expression and different immune cells from ImmuCellAI database. **(C)** Correlation between AP3S1 expression and TAMs/CAFs from TIMER2 database. Red represents positive correlation, blue represents negative correlation, and the darker the color, the stronger the correlation. *p < 0.05, **p < 0.01, ***p < 0.001.

Dysregulation of immune cell infiltration during tumor development and progression can lead to evasion of immune surveillance by tumor cells. Therefore, we used three different methods to assess the relevance of AP3S1 levels to immune cell infiltration in pan-cancer. By analyzing the association between AP3S1 expression and immune cell infiltration using a previous work, we found that AP3S1 expression was positively correlated with infiltration of tumor-associated macrophages (TAMs) and negatively correlated with immune killer cells, such as NK cells and CD8^+^ T cells, in most cancers (Figure 9A). Next, we analyzed the relationship between AP3S1 expression and immune cell infiltration using immune data from the ImmuCellAI and TIMER2 databases, respectively (Figures 9B,C). The clustering heat map showed a positive correlation between AP3S1 and TAMs, which was consistent with the previous results. In addition, we also found that AP3S1 expression was positively correlated with cancer-associated fibroblasts(CAFs) and Tregs. These results revealed that patients with high AP3S1 expression might be in a relatively immunosuppressive microenvironment.

### Immune-Related Genes Analyses

**FIGURE 10:**
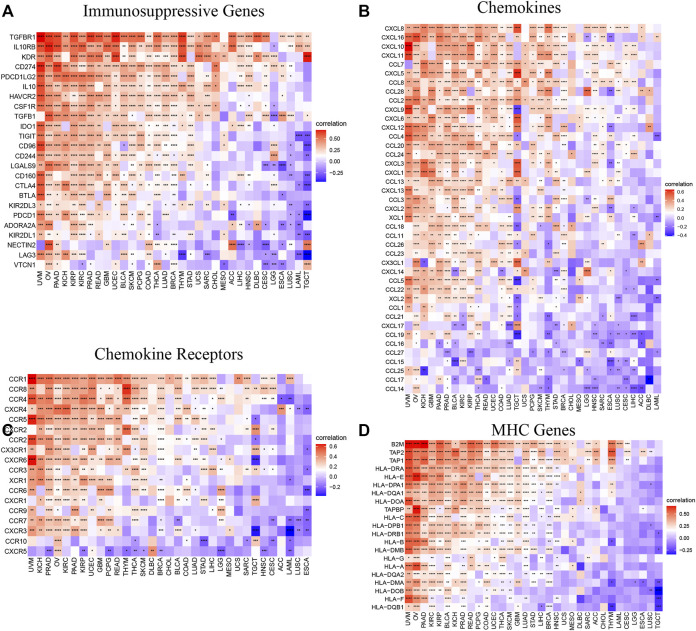
Relationship between AP3S1 expression and that of immune-related genes. **(A)** Immunosuppressive genes. **(B)** Chemokines. **(D)** Chemokine receptors. **(C)** MHC genes. Red represents positive correlation, blue represents negative correlation, and the darker the color, the stronger the correlation. *p < 0.05, **p < 0.01, ***p < 0.001.

To further explore the relationship between AP3S1 and the immunosuppressive microenvironment, we also performed a correlation analysis between immune-related genes and AP3S1. AP3S1 expression was positively correlated with most immunosuppressive genes in pan-cancer, such as CD274 (PD-L1), PDCD1 (PD-1), CTLA4, LAG3 and TIGIT (Figure 10A). We also found that AP3S1 expression significantly and positively correlated with TGFB1 and IL-10 expression in pan-cancer, which in turn were significantly correlated with TAMs/CAFs, and we speculate that this may be a potential mechanism by which AP3S1 affects infiltration of TAMs/CAFs. Finally, we further demonstrated that AP3S1 was closely associated with immunomodulatory genes, including chemokines (Figure 10B), chemokine receptors (Figure 10C) and MHC genes (Figure 10D). Among them, immune-related genes such as CCL2, CCR2, CXCR4, and CCR5 play a role in the recruitment of TAMs in tumor development and progression. TAMs are also able to mediate the immunosuppressive activity of T cells by releasing immune-related genes such as CCL3, CCL4, CCL5, and CCL22. These results suggest that AP3S1 plays an essential role in the process of immunosuppressive microenvironment.

### Tumor Mutational Burden and Microsatellite Instability Analysis

**FIGURE 11:**
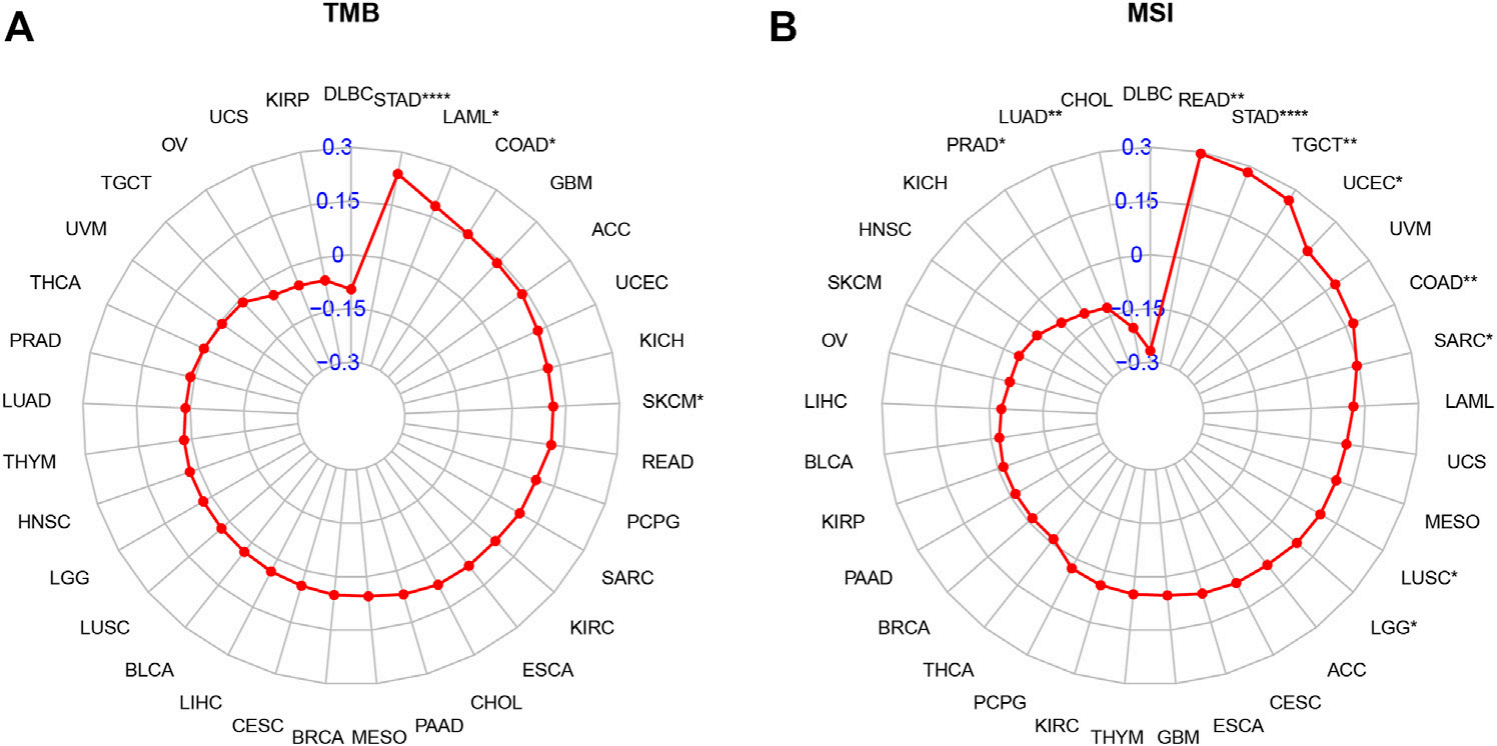
Relationship of AP3S1 expression and tumor mutational burden (TMB), microsatellite instability (MSI). **(A)** Radar map illustrating the relationship between AP3S1 expression and TMB. **(B)** Radar map illustrating the relationship between AP3S1 expression and MSI. The red lines represent correlation coefficients, and blue values represent ranges. Spearman correlation test, *p < 0.05, **p < 0.01, ***p < 0.001.

Tumor mutation burden (TMB) or microsatellite instability (MSI) can predict immunotherapeutic response in different tumor types. Patients with tumors with high TMB or MSI tend to have higher sensitivity to immunotherapy. Therefore, we assessed their relationship with AP3S1 expression in pan-cancer. The results showed a clear positive correlation between AP3S1 expression and TMB in four cancers, including STAD, LAML, COAD and SKCM (Figure 11A). Similarly, AP3S1 expression was correlated with MSI in 10 cancers, positively correlated with READ, STAD, TGCT, UCEC, COAD, SARC, LUSC, and LGG, and negatively correlated with LUAD and PRAD (Figure 11B). These results suggest a potential role for AP3S1 in immunotherapy.

## Discussion

AP3S1 as one of the subunits encoding the adaptor complex AP-3 facilitates the budding of vesicles from the Golgi membrane and may be directly involved in trafficking to lysosomes (Simpson et al., 1997). Although AP3S1 plays an vital role in biological functions, its role in tumors has been neglected in the past. Only a few studies have found a correlation between AP3S1 and tumor progression (Petrenko et al., 2006; Nome et al., 2013; Zou et al., 2021). Here, we extensively analyzed the expression profile and prognostic significance of AP3S1 and explored its potential role in tumor immunology.

Tumor microenvironment (TME) plays a crucial role in tumor development and metastasis (da Silva et al., 2021). A increasing number of evidences support the clinicopathological significance of TME in predicting the survival status and treatment outcome of tumor patients (Binnewies et al., 2018; Thorsson et al., 2018). Recent studies have shown that tumor cells can evade immune surveillance through various mechanisms, especially TAMs and CAFs are remodeled directly or indirectly by tumor cells, thus inhibiting the cytotoxicity of anti-tumor immune cells and subsequently exerting immunosuppressive and tumor promoting effects (Gajewski et al., 2013; Ngambenjawong et al., 2017; Armitage et al., 2021; Cendrowicz et al., 2021). Additionally, tumor cells can also evade immune responses and immunotherapy by utilizing immune checkpoint genes, such as PD-1, PD-L1, and CTLA-4 (Topalian et al., 2012; Barbee et al., 2015).

In our study, we first assessed the expression of AP3S1. The results showed that compared to paracancerous and normal tissues, AP3S1 gene mRNA was highly expressed in 16 types of cancers, including ACC, BRCA, CHOL, COAD, DLBC, HNSC, KIRC, KIRP, LIHC, LUAD, LUSC, PAAD, READ, STAD, THCA, and THYM, whereas low expression was observed in ESCA, GBM, KICH, LAML, LGG, OV, SKCM, TGCT, and UCEC. In addition, gene mutations, DNA methylation, and CNA had important effects on AP3S1 expression in pan-cancer. To determine the prognostic role of AP3S1, we performed UniCox and Kaplan-Meier survival analyses on the TCGA cohort. The UniCox results revealed that AP3S1 was a risk factor for LUAD, GBM, PAAD, KIRP, BRCA, KICH, and LIHC. Kaplan-Meier OS analysis demonstrated that elevated AP3S1 expression predicts that BRCA, GBM, HNSC, KIRP, LIHC LUAD, MESO, PAAD, and UVM patients with a poor OS. These findings suggest that AP3S1 is a potential biomarker to predict the prognosis of tumor patients.

To explore the significance of AP3S1 in cancer immunity, we found that AP3S1 has a broad immunomodulatory function in pan-cancer by using GSEA and GSVA analysis. The correlation of AP3S1 levels with infiltrating immune cells in patients was also assessed. Our results show that AP3S1 expression positively correlated with the level of infiltration of immunosuppressive cells in pan-cancer, such as TAMs and CAFs. In contrast, in most cancers, AP3S1 expression was negatively correlated with immune killer cells, such as NK cells and CD8^+^ T cells. Not only that, we also performed a correlation analysis between immune-related genes and AP3S1. AP3S1 expression was positively correlated with most immunosuppressive genes in pan-cancer, such as the common CD274 (PD-L1), PDCD1 (PD-1), CTLA4, LAG3, and TIGIT. In addition, we found that AP3S1 expression was positively correlated with immunomodulatory genes, including MHC genes, chemokines and chemokine receptors. TAMs are critical players in tumor progression, metastasis and recurrence. They are the most abundant tumor-infiltrating immune cell population in TME. TME is usually classified into two functionally distinct subtypes, the classically activated M1 and the alternatively activated M2 subtypes (Jayasingam et al., 2019). Upon stimulation by a TLR, microbial substrates, IFNγ and CSF2, M1-TAMs secrete cytokines such as IL6, IL12, IL23 and TNFα and express specific M1 markers like MHCII molecules, CD68, CD80, CD86, iNOS, and pSTAT. However, M2-TAMs are the main urban component of TAMs. Upon stimulation by IL4, IL13, IL10, TGFβ and chemokines, M2-TAMs secrete IL4, IL13, IL10, TGFβ, and PGE2 (Cortez-Retamozo et al., 2012; Movahedi and Van Ginderachter, 2016; Dehne et al., 2017). M2-TAMs are able to participate in inflammatory regression and suppress the cytotoxicity of antitumor immune cells, thus allowing tumor cells to evade immune surveillance (Chen et al., 2019; Badmann et al., 2020). The decrease in immune killer cells such as NK cells and CD8 T cells may also increase the ability of tumors to evade immune attack. In recent years, inhibition of TAMs recruitment or retention at primary tumour and metastatic sites has emerged as a new targeted therapeutic approach (Argyle and Kitamura, 2018). Among them, immune-related genes such as CCL2, CCR2, CXCR4 and CCR5 play a vital role in the recruitment of TAMs in tumorigenesis and progression. These genes can be targeted to inhibit tumor growth by suppressing immunosuppressive mechanisms (Pienta et al., 2013; Bonapace et al., 2014; Peña et al., 2015). In addition, TAMs are also able to mediate the immunosuppressive activity of T cells by releasing immune-related genes such as IL10, TGFβ, CCL3, CCL4, CCL5, and CCL22, and by inhibiting genes encoding granzymes, perforins and cytotoxins (Chen et al., 2003; Thomas and Massagué, 2005; Kim et al., 2011). TMB and MSI can be used as predictors of the efficacy of immune checkpoint inhibitors (Samstein et al., 2019). In this study, we demonstrated that AP3S1 expression was associated with TMB in four cancer types and MSI in ten cancer types. These findings implied that high expression of AP3S1 is associated with an immunosuppressive microenvironment in pan-cancer, providing a potential drug target for tumor immunotherapy.

However, we must acknowledge some limitations in the current study. First, all analyses were performed on data from public databases only, and all samples used in our study were obtained retrospectively. Therefore, inherent case selection bias might have affected the results. Extensive prospective and *in vivo* and *in vitro* experimental studies are needed to confirm our findings. Although our study was analyzed and validated using many patient samples from different databases, it opened new perspectives and outlooks for cancer treatment. Based on this study, researchers could aim to understand the outstanding potential of AP3S1 in tumor immunity, conduct various experiments to explore it in-depth and contribute to cancer treatment.

In conclusion, our study suggests that AP3S1 is a potential prognostic biomarker and therapeutic target for pan-cancer. High expression of AP3S1 may contribute to the formation of an immunosuppressive microenvironment in tumors. Targeting AP3S1 could be a novel approach for tumor immunotherapy.

## Data Availability

The datasets presented in this study can be found in online repositories. The names of the repository/repositories and accession number(s) can be found in the article/Supplementary Material.
