# Supplementary figures and images for: AP3S1 is a Novel Prognostic Biomarker and Correlated With an Immunosuppressive Tumor Microenvironment in Pan-Cancer
Source: Front Cell Dev Biol. 2022 Jul 8;10:930933. doi: 10.3389/fcell.2022.930933 (PMC9304770; doi:10.3389/fcell.2022.930933)

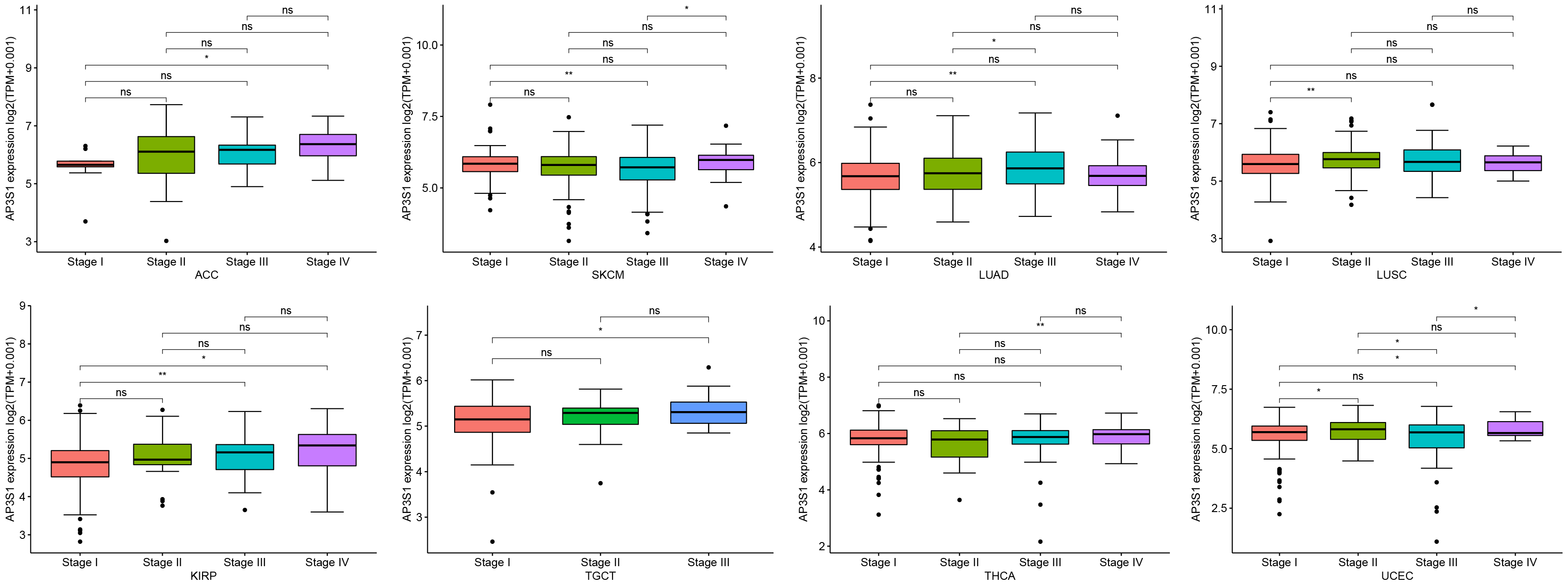

Supplement: Supplementary file 1 [file Image2.TIF]

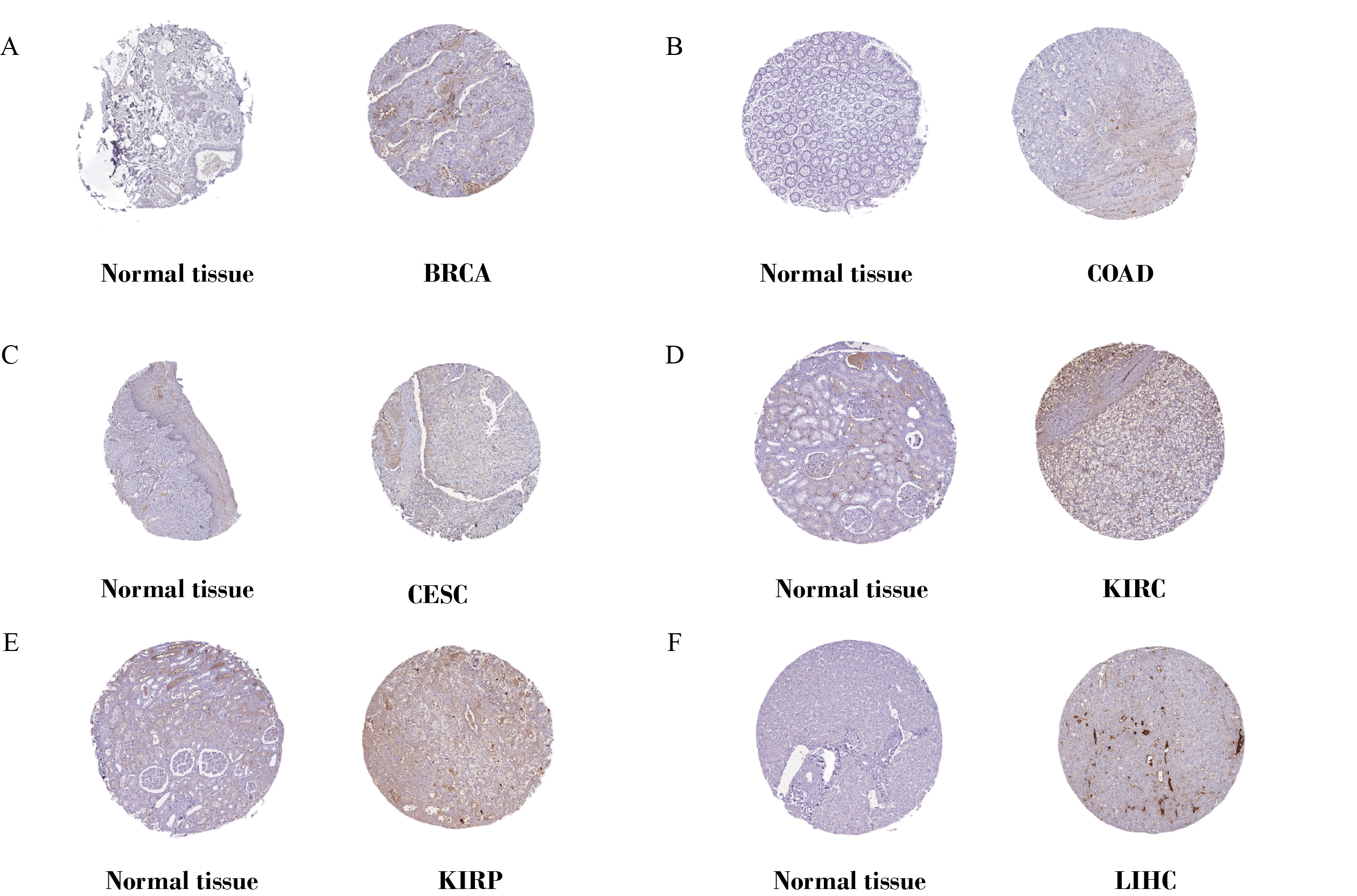

Supplement: Supplementary file 2 [file Image1.TIF]
